# Instrument-Independent MS/MS Database for XQQ Instruments: A Kinetics-Based Measurement Protocol

**DOI:** 10.6028/jres.094.027

**Published:** 1989

**Authors:** Richard I. Martinez

**Affiliations:** National Institute of Standards and Technology, Gaithersburg, MD 20899

**Keywords:** CAD, CBRIS, characteristic branching ratios of ionic substructures, collisionally-activated dissociation, database, ion-molecule kinetics, measurement protocol, MS/MS, NIST-EPA International Round Robin, spectral library, standardization, tandem mass spectrometers, XQQ instruments (QQQ, BEQQ, etc.)

## Abstract

A detailed kinetics-based measurement protocol is proposed for the development of a standardized MS/MS database for XQQ tandem mass spectrometers. The technical basis for the protocol is summarized. A CAD database format is proposed.

## 1. Introduction

Tandem mass spectrometry (MS/MS) is widely used for structure elucidation and for the analysis of multicomponent mixtures [1]. The analysis makes use of the collisionally-activated dissociation (CAD) of “parent” ions. A parent ion may be a molecular radical cation, a protonated molecule, or a “progeny” fragment ion (daughter, granddaughter, etc. produced by the fragmentation of a larger precursor parent ion). The MS/MS technique consists of three steps: (i) a parent ion is mass selected by the first mass analyzer (designated MS-I); (ii) the selected parent ion collides with a target gas which is located within a reaction region between MS-I and the second mass analyzer (designated MS-II); and (iii) the undissociated parent ions and the progeny fragment ions which were produced within the reaction region are channeled into MS-II for mass analysis. MS/MS instruments thus produce a CAD spectrum of each initially-selected parent ion.

Reference [1] provides an excellent up-to-date review of the capabilities and advantages of the MS/MS technique. Among the advantages enumerated in [1] for MS/MS are:

- the functional group specificity of neutral loss experiments (for which there is no analogue in GC/MS) provides the unique ability to rapidly screen unknown mixtures for compound classes (e.g., nitroaromatic cations can be inferred by their loss of NO).
- the ability to perform very rapid analyses of targeted compounds in complex mixtures based on unique progeny ions (especially when “soft” ionization techniques are used) or on neutral loss experiments [e.g., the nitroaromatics screened above in (a)], thus avoiding the need to identify every component.
- very low limits of detection (≤ppt; better than a single stage of mass spectrometry) can be attained with enhanced signal/noise ratios because of the elimination of “chemical noise” [1].
- unstable or extremely reactive species, which cannot be studied directly by optical spectroscopies, NMR, or GC/MS, have been studied directly by MS/MS (e.g., radical trapping of polyatomic free radicals; neutralization-reion-ization mass spectrometry [1]; etc.).

In principle, standard CAD spectra of a variety of ions (fragment ions, molecular ions, protonated molecules, etc.) could be generated and collected as reference libraries, to be used for comparison against unknown spectra in a manner analogous to the use of reference libraries in the data handling systems of ordinary electron impact mass spectrometers. Further, it should be possible to infer the identity of an unknown molecule by identifying the ionic substructures of fragment ions generated in its CAD spectrum. However, to date reference libraries of CAD spectra have not been collected because of a lack of standardization of operating conditions of MS/MS instruments [1].

This paper addresses the standardization of one of the major classes of commercially-available analytical MS/MS instruments, the so-called “XQQ” tandem mass spectrometers (there are currently more than 400 XQQ MS/MS instruments worldwide, representing a capital investment in excess of $170 million). XQQ instruments have three components: XX Q2 Q3. X1 designates the first mass analyzer, which can be a quadrupole mass filter (represented by a Q), a reversed-geometry magnetic/electrostatic sector instrument (represented by BE), etc. Q2 designates a quadrupole mass filter operated with only radiofrequency (rf) potentials [no direct current (dc) potentials are used]. Q2 contains the target gas, and provides efficient ion containment of parent ions and of progeny fragment ions. Q3 stands for the second mass analyzer, a quadrupole mass filter.

Because XQQ instruments (QQQ, BEQQ hybrid, etc.) are complex ion-optical devices [2]–[9], the observed spectra can be extremely dependent on the experimental parameters used during the analysis. That is, one observes instrument-dependent CAD spectra. This was clearly demonstrated in a 1983 international round robin [10] wherein very different CAD spectra were observed for the same molecule. That is, the relative intensities measured in different QQQ instruments for any given pair of progeny ions differed by factors ranging into the hundreds, even though the same *nominal* operating conditions were supposedly used in each of the QQQ instruments. Therefore, until now it has not been possible to use a CAD spectrum of a given species in one XQQ instrument to identify and quantitate that same species in a different XQQ instrument.

To develop an instrument-independent MS/MS database (or library) for XQQ instruments, instrument users must be able to tune their instruments to obtain undistorted, dynamically-correct (i.e., instrument-independent) representations of any reactions studied within such instruments [9]. That is, the measurements must be substantially free from kinetic interferences (viz., every effort must be made to ensure a well-defined gas target and to prevent back reactions, impurity reactions, scattering losses, fringing fields, mass discrimination, etc.). The branching-ratio measurements must be precise and accurate (±10%).

Recent work from this laboratory has explored the prerequisite conditions for obtaining instrument-independent dynamically-correct branching ratios for the CAD of ions within XQQ instruments [9]. In this paper a kinetics-based measurement protocol is described for the determination of standard CAD spectra within XQQ instruments. The technical basis for the protocol is summarized. Table 1 includes a typical CAD spectrum measured using this protocol.

This protocol can also be used for the development of a standardized MS/MS database for XQQ instruments. One of the goals of this paper is to promote discussion about the database format best suited to the needs of the analytical mass spectrometry community. The spectrum of table 1 illustrates the database format proposed here. The precepts of the protocol are also applicable to other types of tandem mass spectrometers which have strong focusing properties (e.g., quadrupole-hexapole-quadrupole MS/MS), so long as the energy range used for the pertinent ion chemistry is the same as for XQQ instruments.

The kinetics notation and nomenclature developed in reference [9] are used throughout this paper.

## 2. General Background

As has been demonstrated [11]–[16], dynamically-correct branching ratios can be measured in XQQ instruments when the key MS/MS instrument parameters [9] are properly selected to account for reaction-induced mass discrimination [9] within Q2 and the intrinsic mass discrimination within the Q3 mass analyzer.

The systems studied in this laboratory include charge transfer and dissociative charge transfer reactions, and CAD.

Charge Transfer:

$${\text{Ne}}^{+}+\text{Ne}\to {\text{Ne}+\text{Ne}}^{+}$$

[11],

$${\text{Ar}}^{+}+\text{Ar}\to {\text{Ar}+\text{Ar}}^{+}$$

[12],

$${\text{and Ar}}^{+}+N_{2}\to \text{Ar}+{N_{2}}^{+}$$

[13]

Dissociative Charge Transfer:

$$\begin{array}{l} {N_{2}}^{+}+{\text{SF}}_{6}\to N_{2}+{{\text{SF}}_{x}}^{+}+\left(6-x\right)F\\ \left(\text{where}\;x=1-5\right) \end{array}$$

[14]

CAD:

$${\left({\text{CH}}_{3}\right)}_{2}{\text{CO}}^{+}\overset{\text{Ar CAD}}{\to }{\text{CH}}_{3}{\text{CO}}^{+}+{\text{CH}}_{3}$$

[15]

$${\text{CH}}_{3}{\text{CO}}^{+}\overset{\text{Ar CAD}}{\to }{{\text{CH}}_{3}}^{+}+\text{CO}$$

[16]Note that for collision energies *E*_LAB_≃1 – 200 eV in the Laboratory (LAB) frame of reference, charge transfer reactions are experimentally equivalent to a “worst-case” CAD reaction system because they take place at large impact parameters with near-zero momentum transfer. That is, the product ions of a charge transfer reaction are formed essentially at rest (thermal energies) within Q2 [17]–[20].

The studies in our laboratory led to the development of a kinetics-based measurement protocol for the generation of standard XQQ spectra. Using the protocol, one can obtain, for the first time, accuracy and precision for CAD measurements within XQQ tandem mass spectrometers. A round robin exercise, the NIST-EPA International Round Robin [21], was organized to ascertain which commercially-available XQQ instruments are capable of generating dynamically-correct (i.e., instrument-independent) spectra. Appendix 1 contains the actual round-robin protocol which was disseminated to 22 laboratories worldwide (including the six XQQ instrument manufacturers). Analysis of the round robin data from six participants indicates that at least 50% of the QQQ instruments which have been sold and are currently in the field can provide such standard spectra [21].

Instruments which can generate dynamically-correct results with the round robin protocol of Appendix 1 can be used to develop a generic, standardized CAD spectral database (or library) based on Characteristic Branching Ratios of Ionic Substructures (CBRIS) (analogous to the use of group frequencies in infrared spectroscopy; see section 5.1). Hence, members of the analytical mass spectrometry community who have such instruments could use the kinetics-based measurement protocol detailed in this paper to generate and contribute instrument-independent CAD spectra of species studied during the course of their work. Contributed CAD spectra (to be sent to this author) would be included in the NIST-EPA Standardized CAD Database currently being developed in our laboratory. The latter would be disseminated by NIST to the analytical mass spectrometry community. To facilitate the critical evaluation of contributed spectra, a dynamically-correct CAD spectrum of a well-studied “model” compound (e.g., n-butylbenzene) should also be submitted.

## 3. Technical Basis for Protocol

Here we summarize the technical basis for the kinetics-based measurement protocol. For a more detailed treatment, the reader is referred to references [2]–[9].

### 3.1 Kinetics

With reference to the following general reaction sequence:

$$\begin{array}{l} A^{+}+B\to C^{+}+S\;\left(\propto \sigma \right)\\ \;\to D^{+}+T\;\left(\beta \sigma \right)\\ \;\text{etc}. \end{array}$$

equations (1)–(3) are applicable under pseudo-first order {[B]_0_>>[A^+^]_0_}, *single-collision* conditions for a reaction zone of length *L* wherein the number density of the target gas is [B] and the “target thickness” is [B]*L.* Here *σ* (= ∝ *σ+βσ* + …) is the total cross section for the A^+^+B interaction, and the sum of the branching ratios ∝ +*β* + … is equal to 1.

Reactant Ion Decay:

$$\ln\;Y\equiv \ln\left\{{\left[A^{+}\right]}_{0}∕\left[A^{+}\right]\right\}=\sigma \left[B\right]L$$ (1)

andProduct Ion Formation:

$$\begin{array}{l} \text{ln}\;W_{\propto }\equiv \ln\left\{\propto {\left[A^{+}\right]}_{0}\;∕\left(\propto {\left[A^{+}\right]}_{0}-\left[C^{+}\right]\right)\right\}\\ \;=\sigma \left[B\right]L \end{array}$$ (2)

$$\begin{array}{l} \ln\;W_{\beta }\equiv \ln\left\{\beta {\left[A^{+}\right]}_{0}\;∕\left(\beta {\left[A^{+}\right]}_{0}-\left[D^{+}\right]\right)\right\}\\ \;=\sigma \left[B\right]L \end{array}$$ (3)

etc.In the case of CAD, A^+^ corresponds to the parent ion; B corresponds to the target gas; C^+^, D^+^, etc. are the progeny fragment ions; and S, T, etc. are the neutral fragments complementary to C^+^, D^+^, etc.

Under dynamically-correct conditions, the rate of reactant ion decay equals the rate of product ion formation. That is ln *Y* = ln *W*_∝_ = ln *W_ß_*, etc. Then the product ion intensities C^+^, D^+^, etc. are related to the extent of consumption of the reactant ion {[A^+^]_0_ − [A^+^}by eqs(4),(5), etc.

$$\left[C^{+}\right]=\propto \left\{{\left[A^{+}\right]}_{0}-\left[A^{+}\right]\right\}$$ (4)

$$\left[D^{+}\right]=\beta \left\{{\left[A^{+}\right]}_{0}-\left[A^{+}\right]\right\}$$ (5)

etc.

#### 3.1.1 Dynamically-Correct Representation

If the key MS/MS parameters [9] of an XQQ instrument can be tuned so that data generated by the instrument conform to equations (1)–(5) [viz., so that ∝ + *β* + *γ* + … = 1.00], then the instrument can provide a dynamically-correct representation of any reaction studied within it. That is, by using eqs (4′). (5′), etc., the instrument would provide a measure of the branching ratios equivalent to those that, in principle, would be observed at the scattering center of a molecular beam machine.

$$\propto =[C^{+}]/\left\{{\left[A^{+}\right]}_{0}-\left[A^{+}\right]\right\}$$ (4′)

$$\beta =[D^{+}]/\left\{{\left[A^{+}\right]}_{0}-\left[A^{+}\right]\right\}$$ (5′)

etc.

#### 3.1.2 Kinetics Constraints

The selection of key MS/MS parameter settings is constrained by the kinetics prerequisite for ∝ + *β* + *γ* + … = 1 (see reference [9]). This prerequisite necessitates that:

- each product ion be formed only by the primary reaction (no secondary sources; pseudo-first order, single-collision conditions)
- all ions be detected with equal sensitivity (requires Conversion Gain corrections for each detector; see sec. 4)
- there be no scattering losses because of unreac-tive collisions (must have high ion containment within Q2; approximately 100% collection efficiency for product ions and unreacted projectiles)
- corrections be made for differences in ion containment (transmission) within the Q2Q3 structure.

### 3.2 Instrumental Parameters

To accomplish ∝ + *β* + *γ* + … = 1.00, the key MS/MS parameters must be properly selected to obviate discrimination against product ions because of:

- Reaction-Induced Mass Discrimination (RIMD) within Q2 (refer to sec. 3.2.1),
- Intrinsic Mass Discrimination (IMD) within Q3 (refer to sec. 3.2.2), or
- the kinetic energy of product ions entering Q3 (refer to sec. 3.2.3).

We shall use the terms “RIMD-free” and “IMD-free” to refer to branching ratios ∝, *β*, etc. which have been measured in concordance with the precepts detailed next for (a)–(c). That is, to be RIMD-free and IMD-free, the instrument parameters discussed below in sections 3.2.1–3.2.3 have to be tuned and returned iteratively until ∝ + *β* + *γ* + … ≃ 1.0±0.1.

#### 3.2.1 Reaction-Induced Mass Discrimination (RIMD) Within Q2

The rf amplitudes of Q1, Q2, and Q3 are characterized here by the Mathieu parameters *q*_1_, *q*_2_, and *q*_3_, respectively (for further information, see references [2]–[5]). In this discussion $q_{\text{react}}^{\max}$ and $q_{\text{react}}^{\max}$ are used to represent, respectively, the values of *q*_2_ which correspond to the maximum ion transmission through Q2Q3 for the reactant (projectile) ion of mass *m*_react_ and for each product ion of mass m_prod_. The subscripts “react” and “prod” designate, respectively, the reactant ion A^+^ and the product ion C^+^ (or D^+^, etc.).

Within Q2, *m*_prod_/*m*_react_=*q*_react_/*q*_prod_ [9]. Therefore, low-mass daughters are not detected when *m*_prod_/*m*_react_<*q*_react_/0.908 since ion trajectories are unstable when *q*_2_>0.908 [5]. This means that the signal for each product ion must be measured at its respective $q_{\text{prod}}^{\text{max}}$. For each product ion, its corresponding {[A^+^]_0_ − [A^+^]} must also be measured at that same $q_{\text{prod}}^{\max}$. That is, with reference to eq (4’), ∝ must be determined by measuring [C^+^] and {[A^+^]_0_ − [A^+^]} at the $q_{\text{prod}}^{\max}$ for C^+^; with reference to eq (5’), *β* must be determined by measuring [D^+^] and {[A^+^]_0_ − [A+]} at the $q_{\text{prod}}^{\max}$ for D^+^; etc. This must be done to compensate for the differences in ion containment efficiencies within the Q2Q3 structure.

For example, consider the CAD of a parent ion of *m/z* 196 which fragments to progeny ions of *m/z* 15, *m/z* 42, and *m/z* 86 [5]. In this case oc must be determined by measuring [15^+^] and [196^+^]_0_ − [196^+^]} at the $q_{\text{prod}}^{\max}$ for 15^+^; *β* must be determined by measuring [42^+^] and {[196^+^]_0_ − [196^+^]} at the $q_{\text{prod}}^{\max}$ for 42^+^; etc. The reader may wish to consult reference [5] for a more detailed exposition.

In principle, if XQQ instruments were well behaved, all measurements could be made at the $q_{\text{prod}}^{\max}$ for the progeny ion of lowest mass (*m/z* 15 for the example above [5]). However, because of ion-optical imaging (focusing) problems within the Q2Q3 structure, one usually observes oscillations in the ion intensity as *q*_2_ is varied [5]. Consequently, measurements of [C^+^], [D^+^], etc. and their respective {[A^+^]_0_ − [A^+^]} must be made at the local maxima closest to the $q_{\text{prod}}^{\max}$ of each product ion.

#### 3.2.2 Intrinsic Mass Discrimination (IMD) within Q3

The resolution controls for Q3 must be varied as necessary to compensate for the mass discrimination intrinsic to Q3 {cf. [2]: p. 100, p. 105 (fig. 5.9), and p. 143–144}.

#### 3.2.3 The Kinetic Energy of Product Ions Entering Q3

It is now recognized that in the LAB frame of reference the translational energy of product ions *E*_prod_ is generally related to the collision energy of the projectile (reactant) ion *E*_react_ by:

$$E_{\text{prod}}≃{\left(m_{\text{prod}}∕m_{\text{react}}\right)}^{n}E_{\text{react}},$$ (6)

where *n* ≃ 1 − 2 for CAD processes studied to date [8]. Hence, if *m*_prod_/*m*_react_ < < 1, low-mass daughters exiting Q2 will have low kinetic energies. Consequently, the Q3 rod offset (pole bias) must be set sufficiently low with respect to the Q2 rod offset so that no low-mass, low-energy product ions are denied entry to Q3 by the Q3 potential barrier. On the other hand, the Q3 potential barrier must be sufficiently high to provide adequate resolution within Q3 [8]. The reader may wish to consult references [7] and [8] for a more detailed exposition with reference to the selection of the appropriate Q3 rod offset.

## 4. Kinetics-Based Protocol for MS/MS Measurements

In this section we detail the generic protocol used to measure instrument-independent CAD spectra in XQQ instruments. The reader is referred to the caveats detailed in items 1–9 of the *Precautions* in Appendix 1.

- The protocol of Appendix 1 (for the NIST-EPA International Round Robin) must first be used to validate that any particular XQQ instrument can provide a dynamically-correct (i.e., instrument-independent) representation of ion-molecule reactions. If the instrument cannot pass the protocol (i.e., it cannot provide a dynamically-correct representation), one should not proceed any further until one has eliminated the ion-optical defect(s) so that the instrument can pass the protocol. If the instrument can provide a dynamically-correct representation, then the measurements for Part 1 of the round-robin protocol of Appendix 1 will provide an in situ calibration of the target thickness of the XQQ instrument.
- For all MS/MS measurements, one must use a gas target thickness within the single-collision regime [<2 cm-mtorr (<0.267 cm-Pa) for Ar]. For CAD, use an Ar gas target since it has been demonstrated [22] that the equivalent excitation energy (i.e., the equivalent internal energy imparted to a parent ion) is significantly less for a polyatomic target than it is for a monatomic target. However, one may also use other inert gas targets (He, Ne, Kr, or Xe) as necessitated by the collisional energy requirements since it has been demonstrated that the equivalent excitation energy is the same for the inert gases at any given center-of-mass energy *E*_CM_ [22]–[24] {*E*_CM_ = *E*_LAB_ [*m*_2_/(*m*_1_+*m*_2_)], where *m*_1_ and *m*_2_ are, respectively, the masses of the reactant ion and of the gas target}.
- One must use a *fixed* operating voltage for the ion detector (multiplier, Daly detector, etc.). The fixed voltage should provide the best compromise between the signal-to-noise ratio (*S/N*) of A^+^ and the *S/N* of C^+^, D^+^, E^+^, etc.
- One must make Conversion Gain measurements if an instrument uses analog current measurements. Conversion Gain is the ratio of the electron current output from an electron multiplier relative to the ion current input. The Conversion Gain measurements are used to correct for differences in mass-dependent detector response. If the instrument uses *true* ion pulse counting, Conversion Gain measurements are *not* needed [i.e., ignore instruction (d) and continue with instruction (e)].
  *Warning*: Some instruments use analog current measurements, but report the ion intensities as equivalent ion count rates within their data systems. Such instruments still require Conversion Gain measurements.
  *Note*: If possible, one should adjust the Conversion Gain of the ion detector so that one attains a mass-independent detector response. That is, so that the Conversion Gain is the same for a projectile ion and for its lowest-mass product ion (e.g., for CAD) or highest-mass product ion (e.g., for neutral gain reactions). This might be accomplished, for example, by adjusting the operating voltage of a high-voltage (e.g., 20–30 kV) conversion dynode which is used in conjunction with an electron multiplier. If it is not possible to attain a mass-independent detector response, then one must make corrections for difference in Conversion Gain for each parent ion and for each product ion.
  To measure Conversion Gains, one may use a procedure analogous to that used for instructions 30–39 in Part 2 of the NIST-EPA round robin protocol in Appendix 1. However, to ensure that the most reliable correction factors are obtained, the mass-dependent Conversion Gain measurements would have to be made under actual operating conditions (viz., with the conversion dynode and electron multiplier at high voltages). This might be done by measuring the ion current striking the conversion dynode and relating it to the output current from the electron multiplier. One would have to demonstrate, however, that there is a one-to-one correspondence between the ion current input and the electron current output, independent of ion structure.
  *Note*: For the rest of the instructions which follow, it is presumed that the ion signals correspond to a mass-independent detector response. That is, if necessary, the ion signals have been corrected for differences in Conversion Gain efficiency.
- Adjust the appropriate ion-optical elements (e.g., the Q1 rod offset for QQQ instruments) so that (i) [B]_0_> > [A^+^]_0_ and (ii) the energy spread of the projectile ions entering Q2 is minimized (≤3 eV for 50% of the ions). (Refer to item 3 of the *Precautions* from the NIST-EPA round robin protocol in Appendix 1.) Measure the Q2 stopping curve in a manner analogous to that prescribed in instructions 8–10 of Part 1 of the NIST-EPA round-robin protocol in Appendix 1.
- From the optimum Q2 stopping curve measurements determine *E*_50_ (corresponds to the Q2 rod offset that stops 50% of the reactant ions). Then set the Q2 rod offset equal to *E*_50_ − *E_LAB_* where *E_LAB_* is the requisite collision energy (in LAB coordinates).
- One must use only a “daughter-scan” mode. That is, Q1 is tuned to the peak position which corresponds to the maximum ion intensity at the *m/z* of the projectile ion while Q3 scans over the peak which corresponds to the *m/z* of a product ion. The *q*_2_ must be referenced to *q*_1_ (i.e., to *q*_react_) rather than to *q*_3_ (i.e., to q_prod_). Use a scan window of ca. 4–10 amu so one can see the entire mass peak (baseline-to-baseline).
  *Note*: To ensure consistent measurements for the duration of each experiment, make sure Q1 is staying tuned to the peak position for the projectile ion intensity; this can be done by varying the Q1 mass command to maximize the ion signal being viewed within the Q3 scan window. Use the same *fixed* operating voltage for the ion detector as was used for instruction 4 (c).
- One then adjusts iteratively the resolution parameters for Q3, and the Q3 rod offset as necessary to approximate *∝ + β + γ + …* ≃ 1.0 for the *∝*, *ß, …*, etc. measured in concordance with the precepts detailed in sections 3.2.1–3.2.3. To optimize the precision of all measurements, ion signals should be measured in “back-to-back” units [cf. item 6 (iii) of the *Precautions* from the NIST-EPA round-robin protocol in Appendix 1]. For example, for a fixed [B]L measure:
  [C^+^], [A^+^], and [A^+^]_0_ at the $q_{\text{prod}}^{\text{max}}$ for C^+^;
  [D^+^], [A^+^], and [A^+^]_0_ at the $q_{\text{prod}}^{\text{max}}$ for D^+^; etc.
  *Note*: One must make appropriate corrections for any background contributions to [C^+^], [D^+^], etc. which may be observed in the absence of added target gas. Such background contributions can arise from decompositions of A^+^ (to produce C^+^, D^+^, etc. via CAD or RIF [9]) occurring between the rear of Q1 and the front of Q3. Such background CAD or RIF may be induced by the interaction of A^+^ with plumes of gas emanating from the ion source.
- In practice, instructions (b)–(h) provide IMD-free and RIMD-free estimates for the branching ratios of fragment ions, except for very minor fragment ions adjacent to major ions. The resolution is not adequate for obtaining dynamically-correct estimates for the branching ratios of very minor fragment ions adjacent to major ions. Consequently, the resolution must be increased for each group of ions which contains a minor ion adjaent to a major ion, and complementary measurements must be made for the branching ratios of minor fragment ions within each group. Each group contains only fragment ions which are in close proximity (e.g., *m/z* 13–15), so that the measurements for each ion in the group are still IMD-free and RIMD-free within that group. To relate these individual group measurements to the overall CAD spectrum [viz., to the entire range of masses from the *m/z* of the reactant (parent) ion down to the *m/z* of the lowest-mass product ion], the measurements for each group must be referenced to the IMD-free and RIMD-free estimate for the branching ratio of a major fragment ion within that group. For example, for *E*_CM_≃2−40 eV, the CAD of C_2_H_3_O^+^ (*m/z* 43) from biacetyl produces fragment ions at the following *m/z*: 13, 14, 15, 26, 27, 28, 29. The fragment ions at *m/z* 13 and 14 are minor compared to the major fragment ion at *m/z* 15 (refer to table 1). Therefore, *m/z* 13–15 are measured under well-resolved conditions and referenced to the IMD-free and RIMD-free estimate for the branching ratio of the fragment ion at *m/z* 15. Similarly, the fragment ions at *m/z* 27 and 28 are referenced to the fragment ions at *m/z* 26 and/or 29.
- Repeat (b)–(i) as necessary to accomplish ∝ + *β* + *γ* + …≃1.0±0.1 for each *E*_CM_, as necessitated by the reaction dynamics.

## 5. Proposed Format for MS/MS Database

Table 1 shows a typical CAD spectrum (measured with the protocol of sec. 4 [16]) in the format proposed for presentation of CBRIS.

### 5.1 Advantages

The advantages of this CBRIS database format are:

- The partial cross sections ∝σ, *β*σ, etc. can characterize both known and unknown species (so long as the unknown species contain ionic substructures for which the CAD cross sections and product identities are known). Therefore, it may be possible to assign the structure of an unknown species on the basis of the absolute cross sections σ_ij_, σ_jk_, etc. for the CAD of known ionic substructures i, j, k, etc. That is, this proposed use of CBRIS in MS/MS is analogous to the use of group frequencies in infrared spectroscopy.
- Characterization of an unknown compound by using CBRIS does not require that the compound be in the CBRIS database. By contrast, to characterize an unknown by spectral matching within a “library”, the compound must usually be in the library. In this regard, development of a database of CBRIS for all substructures (or a subset thereof) would be very much more tractable than the development of a library of CAD spectra for all the source compounds that contain all the substructures (or a subset thereof). For example, consider the simplistic analogy where compounds correspond to words, and substructures correspond to letters: more than 500,000 words can be composed with only 26 letters of the alphabet.
- The format is compatible with its use in expert systems. This should facilitate rapid real-time analysis of unknowns within computer-controlled field instruments.
- End users are involved directly in its evolution by using critically evaluated cross sections already in the database and by submitting new cross sections for inclusion in the database.

## 6. Conclusions

A kinetics-based measurement protocol can provide accuracy and precision for CAD measurements within XQQ tandem mass spectrometers. This protocol can be used to develop a dynamically-correct (i.e., instrument-independent) MS/MS database (or library) for XQQ instruments.

## Figures and Tables

**Figure 1:**
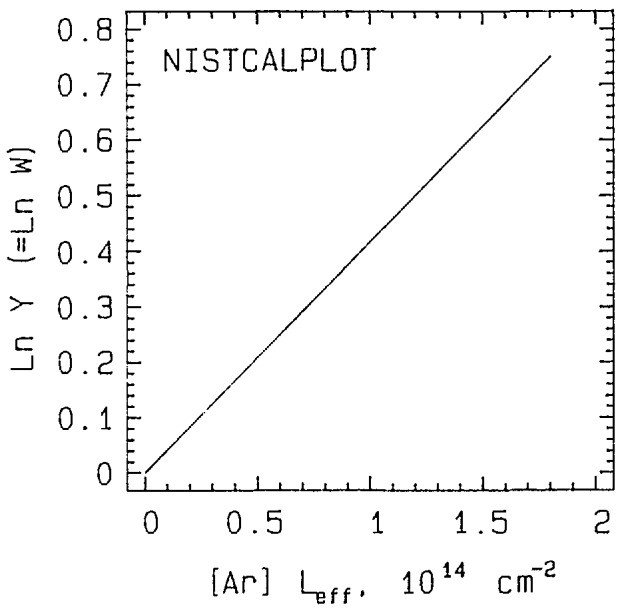
Calibration curve of ln *Y* (=ln *W*) vs effective target thickness [Ar]*L*_eff_.

**Table 1:** Proposed CBRIS^1^ database format^2^,^3^

Parent Ion: Source Compound: Ionization Mode:	C_2_H_3_O^+^(*m*/*z*43)^4^,^5^ 2,3-butanedione (99.9%) 70 eV electrons	Notes: 9, 99, 999^6^

*E*_CM_ (eV)	*σ* (Å^2^)	∝ (13^+^)	*β* (14^+^)	*γ* (15^+^)	*δ* (26^+^)	*ϵ* (27^+^)	*ζ* (28^+^)	*η* (29^+^)
2.4	15	0	0	0.999	0	0	0	0.0010
	[20]			[2]				[50]
19.3	22	0.0127	0.0493	0.915	0.0101	0.0013	0.0005	0.0114
	[10]	[15]	[6]	[2]	[7]	[10]	[35]	[7]
38.6	21	0.0227	0.0750	0.839	0.0436	0.0055	0.0055	0.0086
	[10]	[10]	[6]	[2]	[6]	[20]	[20]	[15]

1 CBRIS = C haracteristic B ranching R atios of I onic S ubstructures.

2 For this example we show the branching ratios (∝ − *η*) vs the center-of-mass collision energy (*E*_CM_) for the CAD of (C_2_H_3_O^+^) from the source compound biacetyl [16].

3 The numbers in the square brackets represent the maximum uncertainty in the cross section *σ* and in the branching ratios (∝ − *η*), expressed as a percentage of each σ and of each branching ratio {e.g., for biacetyl at *E*_CM_ = 2.4 eV, the maximum uncertainty in *y* is ±([2%]/100)γ; i.e., γ=0.999±0.02}.

4 A reference citation would be provided for each CAD spectrum to identify the source of the data.

5 For the CAD of any given parent ion (e.g., C_2_H_3_O^+^), appropriate corrections must be made for contributions from the concurrent CAD of isobaric ions (e.g., C_3_H_7_^+^), regardless of their source. The isobaric ions may be co-produced in the ion source (i) from the source compound (e.g., ionization of CH_3_C(O)C_3_H_7_ will produce both CH_3_CO^+^ and C_3_H_7_^+^) and/or (ii) from a neutral impurity in a source compound (e.g., for a butanol impurity in a 2-butanone source compound, the butanol generates C_3_H_7_^+^, while the 2-butanone generates CH_3_CO^+^). Fortunately, the CAD spectra of CH_3_CO^+^ and C_3_H_7_^+^ are easily distinguishable [16]. That is, for the CAD of C_3_H_7_^+^, C_2_H_3_^+^ (*m/z* 27) is the major CAD fragment for *E*_CM_≃2−80 eV. By contrast, for the CAD of C_2_H_3_O^+^, C_2_H_3_^+^ is not produced at *E*_CM_ = 2.4 eV, and is only a very minor fragment for *E*_CM_ > 2.4 eV. Unfortunately, even minor impurities can contribute disproportionately to the CAD spectrum of a source compound because of differences in the CAD dynamics of isomeric and/or isobaric ions. Because of this problem, it is advisable that both the source compound and the target gas be of high purity (>99.95%). Otherwise, the impurities must be characterized so that appropriate corrections can be made for their contribution to the observed CAD spectrum.

6 The *Notes* field would be used to refer a user of the database to any information of special significance about the parent ion (e.g., structure of the ion, etc.). The notes enumerated in all the *Notes* fields of the CBRIS database would be collected together in a separate “Notes Appendix”. For the example given here, Notes 9, 99, and 999 would be found in the Notes Appendix, and might contain the following types of information.

*Note 9*: For a given *E*_CM_, the branching ratio for each fragment ion is substantially the same for all CH_3_CO—X source compounds (e.g., biacetyl, acetone, acetophenone, etc.) [16]. The reactant ion entering Q2 appears to be pure CH_3_CO^+^ in every case [16].

*Note 99*: The energy dependence (magnitude and direction) of the branching ratios is distinctly different for the isobars C2H_3_O^+^ and C_3_H_7_^+^ [16]. Hence, one can readily distinguish C_2_H_3_O^+^ from C_3_H_7_^+^.

*Note 999*: One can readily distinguish ethanol, oxirane, and cis-2,3-epoxybutane from each other, and from CH_3_CO—X source compounds on the basis of the energy dependence of the branching ratios for the CAD of C_2_H_3_O^+^ [16].

## Appendix 1. NIST-EPA Round Robin for XQQ Instruments (QQQ, BEQQ, etc.): A Test Protocol to Assess Which Instrument Designs Provide Instrument-Independent (Dynamically-Correct) Performance

*Note*: This appendix is the full text of the actual document which was sent to, and was used by, the participants in the NIST-EPA International Round Robin. The round-robin results have been published elsewhere [21]. *This document may be photocopied. Prospective participants may submit test data to the author anytime for this ongoing evaluation project.*

This round robin is being coordinated by Dr. Richard I. Martinez of the U.S. National Institute of Standards and Technology (NIST). It is sponsored by the Environmental Monitoring Systems Laboratory (EMSL) of the U.S. Environmental Protection Agency (EPA).

*Address Inquiries/Comments to*:

Richard I. Martinez, A260 Chemistry Bldg.
NIST, Gaithersburg, MD 20899 USA
Telephone: (301) 975-2516
FAX: (301) 975-2128

### Objective

To assess which XQQ instrument designs can be used to generate a generic, instrument-independent MS/MS database. To do so, an XQQ instrument (QQQ, BEQQ, etc.) must provide a dynamically-correct (instrument-independent) representation of any ion-neutral interaction (e.g., CAD, etc.) studied within its Q2Q3 structure. The prerequisites for obtaining dynamically-correct branching ratios within XQQ instruments have been detailed in reference [9].

### Data Analysis

To ensure consistency, all data will be analyzed by the round-robin coordinator. The participants will provide only “raw data” on the forms provided with the test protocol. To perform the analysis, the raw data will have to include information about such technical specifications as field radius, rf frequency, etc. Please submit one completed copy of the *Questionnaire* for each XQQ instrument included in the round robin.

### Confidentiality

All information provided by participants will be confidential. No proprietary information will be divulged. No participant or instrument manufacturer will be identified (the data files will be encrypted with code letters, numbers, and symbols known only to the round-robin coordinator). All test results will be discussed only in generic terms.

### Summary of Test Protocol

The test protocol is based on (i) the concepts detailed in reference [9], and (ii) the use of the ion-neutral reaction kinetics of references [12] and [14].

The following is a synopsis of the two major components of the test protocol. The nomenclature used herein is defined in references [9], [12], and [14].

#### Part 1

##### Purpose

- To determine if the reaction kinetics are well controlled (i.e., no back reactions, no scattering losses, minimal fringing fields, no mass discrimination, well-defined gas target, etc.) within each XQQ instrument. The symmetric charge transfer reaction ^36^Ar^+^ + ^40^Ar→^36^Ar + ^40^Ar^+^ is used (see reference [12]). In this case there is no reaction-induced mass discrimination (defined in reference [9]).
- To calibrate the response of the pressure sensor closest to the participant’s collision region in terms of the effective target thickness [Ar]*L*_eff_ within the collision region (*L*_eff_ is the effective pathlength of the complex oscillatory trajectory traversed by a projectile ion through the CAD target gas within Q2). This is done to ensure all participants use the same effective target thickness measurements.

##### Glossary

Here we describe some terms used below.

NISTCALPLOT	A calibration curve of In *Y* (=ln *W*) vs effective target thickness [Ar]*L*_eff_. NISTCALPLOT (fig. 1) is included below for use with the test protocol.
P/XQQ	The nominal pressure indicated by some sensor located closest to the participant’s collision region (see *Precaution* 8).
ARLEFF/LNY	The target thickness [Ar]*L*_eff_ which is derived from NISTCALPLOT for a corresponding ln *Y* value measured by a participant for a given P/XQQ.
ARLEFF/LNW	The target thickness [Ar]*L*_eff_ which is derived from NISTCALPLOT for a corresponding ln *W* value measured by a participant for a given P/XQQ.
XQQCALPLOT	A graph containing a plot of ARLEFF/LNY vs P/XQQ and a plot of ARLEFF/LNW vs P/XQQ.

##### Method

Using several different pressures (P/XQQ) of Ar target gas, each participant measures ln *Y and* ln *W* for the reaction ^36^Ar^+^ + ^40^Ar→^36^Ar + ^40^Ar^+^. NISTCALPLOT is then used to convert the participant’s ln *Y* and ln *W* measurements to the corresponding target thicknesses ARLEFF/LNY and ARLEFF/LNW, respectively, These data are then used to prepare the calibration graph XQQCALPLOT, If the kinetics are well controlled within the participant’s XQQ instrument, then the two plots of ARLEFF/LNY vs P/XQQ and ARLEFF/LNW vs P/XQQ should be superimposable.

#### Part 2

##### Purpose

To assess how well one can correct for reaction-induced mass discrimination and for strong ion-optical imaging (focusing) problems within the Q2Q3 structure (i.e., how well one can control the key MS/MS parameters in different XQQ instrument designs) (see reference [9] for further information).

##### Method

Each participant measures ln *Y and* ln *W* for the reaction N_2_^+^ + SF_6_→N_2_ + SF_5_^+^ as a function of the pressure P/XQQ of the SF_6_ gas target (cf. reference [14]).

### Data Format

Different types of data are requested within the test protocol. Most of the test data to be provided by the participants will consist of “filling-in-the-blanks” in the spaces provided with the test protocol.

For laboratories with more than one XQQ instrument participating in the round-robin test, please submit a separate set of data for each XQQ instrument (use photocopies of this test protocol document to record each separate set of data).

#### Precautions

##### 1. Manual vs Automated Control

Some very sophisticated XQQ systems have automated (“intelligent”) self-tuning capabilities (e.g., mass calibration, etc.), automated instrument control (e.g., “programmed ion optics”, MS/MS mass scan modes, etc.) and automated data acquisition, etc.

To provide the best possible assessment of instrument *Hardware* design (without obfuscation by software artifacts), and to ensure consistency among the data submitted, participants must *not* use any of the automated capabilities.

Instrument control and data acquisition should be as close to “manual” control as is possible with your instrument. You may use a “hybrid” control system wherein you are “manually” controlling the parameters [e.g., programmable “soft-knobs” (EXTREL^1^) or “tuning tables” (Finnigan TSQ70) are used to vary the dc voltage which is being applied to a lens via a digital-to-analog converter (DAC)]. However, use only a single “tuning table” with “flat” (constant) parameter settings; don’t use “programmed ion optics”. Please mark the top of the first page of the submitted data with the following label: *Manual Instrument Control and Data Acquisition.*

However, for any one XQQ instrument being tested, participants are welcome to submit a *second* set of data to assess possible artifacts in the instrument control and data acquisition software. Please mark the top of the first page of the submitted data with the following label: *Automated Instrument Control and Data Acquisition.*

If your system has only automated capabilities (i.e., there is absolutely no way that it can be made to function in a manual or hybrid mode of operation for instrument control and data acquisition), please mark the top of the first page of the submitted data with the following label: *Non-Manual Instrument Control and Data Acquisition.*

##### 2. Actual Voltage Measurements

The actual voltages of all essential ion-optical elements [rod offset (pole bias) of Q1, Q2, Q3; lens potentials; etc.] must be measured with a voltmeter at the appropriate measurement test points closest to each ion-optical element. Consult the manufacturer for the location of each test point and the method of measurement that you should use to ensure correct values (e.g., to measure actual rod offsets, you may have to disconnect the inputs for mass command and/or resolution control and/or *∆M* control). Please make actual voltage measurements to validate any voltage information provided by a computer system via an analog-to-digital converter (ADC).

##### 3. Q1 Rod Offset and Projectile Energy Distribution

To be kinetically meaningful, participants must ensure:

- that [B]_0_ > > 100[A^+^]_0_ (in molecules cm^−3^ within Q2; ion intensity data are converted to the approximate ion concentrations with reference to the defining aperture which provides entry into Q2),
- that the measured reactivity can be attributed to a well-characterized state of the projectile ion A^+^, and
- that the translational energy distribution of the projectile ion entering Q2 is reasonably narrow (cf. Q2 stopping curve data in references [12] and [14]). [Unfortunately, narrowing the energy distribution of the projectile ion can produce ion-optical imaging (focusing) problems within Q2 if the XQQ instrument has restrictive interquadrupole apertures (see reference [9]).]

For QQQ instruments, tasks (a)–(c) can be accomplished by over-resolving Q1 (i.e., increasing the resolution of Q1 to produce a sharp, narrow peak) and by raising the Q1 rod offset close to the ion source potential. The effect of these actions is to: (i) discard a large fraction (>90%) of A^+^, (ii) delay the transit of A^+^ through Q1 (this provides sufficient time for excited electronic states of the N_2_^+^ used for *Part 2* to be quenched by radiative transitions prior to their entry into Q2), and (iii) reduce the energy spread of the ions exiting Q1 by cutting out low-energy ions. Unfortunately, rf-pickup within each quadrupole mass filter tends to broaden the energy distribution of the projectile ion as it traverses Q1, Q2, and Q3. Therefore, a high-energy tail is usually observed.

##### 4. Q3 Rod Offset

To achieve ln *W*=ln *Y*, one must have very high ion-collection-efficiency (≃100%). Consequently, the Q3 rod offset must be biased very *negatively* with respect to the Q2 rod offset to draw ions out of Q2. This will result in very broad, poorly resolved mass peaks (reference [8] shows examples of how the Q3 rod offset affects peak shapes). Throughout each *Part* of the test protocol, you must keep the Q3 rod offset biased as negatively as necessary so that [C^+^]≃[A^+^]_0_ − [A^+^].

##### 5. Tuning q_2_

The rf amplitude of Q1, Q2, and Q3 is characterized here by the Mathieu parameters *q*_1_, *q*_2_, and *q*_3_, respectively (for further information, see references [2]–[5]). Some instruments have a separate rf/dc generator (quadrupole controller/power supply) for each of the three quadrupole rod assemblies (Q1, Q2, and Q3); some do not. Some can reference *q*_2_ to *q*_3_ *and/or* to *q*_1_ (i.e., one can vary *q*_2_/*q*_1_, or *q*_2_/*q*_3_ from 0–1; in some instruments the ratio is fixed at *q*_2_/*q*_1_=0.5 or *q*_2_/*q*_3_=0.5). Some instruments reference *q*_2_ to the ion transmission cut-off which occurs at *q*_2_≃0.908 [this latter mode of tuning is somewhat risky; the rf-pickup within Q2 can make it difficult to distinguish the true cut-off at *q*_2_≃0.908 from the small, but significant contribution of ions in the high energy tail which are transmitted through Q2Q3 even though the nominal (apparent) value is *q*_2_>0.908].

In general, tune *q*_2_ by following the recommendations of the manufacturer of your XQQ instrument (please complete the questionnaire regarding modes of operation for your XQQ instrument). However, to ensure experimental consistency among participants, please validate your *q*_2_ values relative to *q*_1_ and/or *q*_3_ (i.e., remember that whenever Q1 or Q3 are operated in a well-resolved mode and are tuned to any *m/z*, then the mass peak position corresponds to *q*_1_≃0.706 or *q*_3_*≃*0.706 for that *m/z*).

One can accomplish the validation of *q*_2_ values with a simple potentiometer divider circuit if the instrument can be operated in a manual or hybrid mode. For example, in some instruments a *Mass Command* input dc voltage (typically 0–10 V) is used to control the rf amplitude of a quadrupole mass filter. This 0–10 V is usually supplied by a DAC which may be controlled by a computer via a programmable soft-knob. Here we use MC1, MC2, and MC3 to designate, respectively, the 0–10 V *Mass Command* input voltages for Q1, Q2, and Q3. Then the divider potentiometer can be used to vary *q*_2_/*q*_1_ (≃MC2/MC1) or *q*_2_/*q*_3_ (≃MC2/MC3). Consult your manufacturer for the location of the inputs analogous to MC1, MC2, and MC3.

If the manufacturer asserts there is absolutely no possible way to vary *q*_2_ within your instrument (i.e., one cannot vary *q*_2_/*q*_1_ or *q*_2_/*q*_3_), please indicate how *q*_2_ is controlled by the manufacturer (i.e., does the manufacturer use a fixed value of *q*_2_/*q*_1_ or *q*_2_/*q*_3_≃0.5, or ?). Also, please mark the top of the first page of the submitted data with the following label: *q*_2_ *Not Varied.*

##### (i) Mode of Operation

Use only a “daughter-scan” mode [i.e., *q*_2_ referenced to *q*_3_; Q1 tuned to the peak position which corresponds to the maximum ion intensity at the *m/z* of the projectile ion; Q3 scanning over the peak which corresponds to the *m/z* of the product ion (use a scan window of ca. 4–10 amu) so you can see the entire mass peak (baseline-to-baseline)].

*Note*: To ensure consistent measurements for the duration of each experiment, make sure Q1 is staying tuned to the peak position for the projectile ion intensity; this can be done by varying the Q1 mass command to maximize the ion signal being viewed within the Q3 scan window.

##### (ii) Signal and Background Measurements

For the reasons discussed in 1 above, all ion signal intensities must be acquired *manually.* To do so, you must use a display which shows the *complete* mass spectral peak shape for a single mass peak (i.e., baseline-to-baseline display of ion intensity vs *m/z* for a single mass peak). For this, you can use either an external oscilloscope connected to the output of your detector chain, or whatever mass spectral display your system provides on its CRT.

For each mass peak, use arbitrary units (e.g., CRT divisions) to measure the ion intensity at the peak. However, report the ion intensities in relative units by making appropriate corrections for differences in amplifier gain, scope attenuation, etc. [e.g., an ion intensity of *1.00 relative units* could correspond to (a) 1 CRT division with an amplifier gain of × 1 and a scope attenuation of × 1, etc. or (b) 10 CRT divisions with an amplifier gain of × 100 and a scope attenuation of × 10].

For the measurement of *background* alone, the ion intensity is measured at the mass peak position in the absence of the ion of interest. For the measurement of *signal+background*, the ion intensity is measured at the mass peak position in the presence of the ion of interest.

*Note*: Please follow the following guidelines to ensure the ion intensity measurements are kinetically meaningful, and to minimize long-term variations in gas flows, detector response, etc. Here source gas refers to the gas introduced into the ion source, and target gas refers to the gas introduced into Q2.

- Before making any ion intensity measurements in Part 1 or Part 2, allow sufficient “warm-up” time to ensure all instrumental components, ion source, gas flows, etc. have stabilized.
- Leave *everything* (source gas, ionizer filament, etc.) turned on at all times for the duration of all measurements in any one Part (the target gas is the only thing that should be turned on and off during the measurements).
- If possible, use a high-quality toggle valve or solenoid shut-off valve to turn the target gas on/off. (The shut-off valve should be located between the Q2 collision region and a high-precision needle valve). Thus, the target gas flow can be reset reproducibly to any given P/XQQ by presetting the needle valve. This will help to ensure high-accuracy “back-to-back” units for ln *Y*/ln *W* measurements.
- To make measurements of the *background* intensity at any *m/z*, block the Projectile Ion Beam (PIB). To do so, raise to a high positive voltage the potential of any ion-optical element (e.g., the rod offset of Q1 or Q2).

The following abbreviations are used throughout the protocol:

PIBOFF = Projectile Ion Beam OFF (projectile ion beam blocked; no ions reach the detector)
PIBON = Projectile Ion Beam ON (projectile ion beam unblocked; ions enter Q2)
TGOFF = Target Gas OFF (not flowing into Q2)
TGON = Target Gas ON (flowing into Q2)

##### (iii) In Y and ln W Measurements

All ln *Y* and ln *W* data should be collected in *back-to-back units* to minimize long-term variations in gas flows, detector response, etc. For example, with reference to equations (1) and (2), one back-to-back unit would consist of items (a)-(g) below. Here $q_{\text{react}}^{\text{max}}$ and $q_{\text{prod}}^{\text{max}}$ represent, respectively, the values of *q*_2_ which correspond to the maximum ion transmission through Q2Q3 for the reactant (projectile) ion A^+^ and for the C^+^ product ion. Note that $q_{\text{react}}^{\text{max}}$ and $q_{\text{prod}}^{\text{max}}$ must be known prior to making the ln *Y* and ln *W* measurements.

- tune *q*_2_ to $q_{\text{react}}^{\max}$ for A^+^.
- PIBOFF/TGOFF: measure background signal at *m/z*(A^+^).
- PIBON/TGOFF: measure [A^+^]_0_ at *m/z*(A^+^).
- PIBON/TGOFF: tune *q*_2_ to $q_{\text{prod}}^{\max}$ for C^+^; measure background signal at *m/z*(C^+^).
- PIBON/TGON: measure [C^+^] at *m/z*(C^+^); tune *q*_2_ to $q_{\text{react}}^{\max}$; measure [A^+^] at *m/z*(A^+^).
- repeat (c).
- repeat (b).

##### (iv) Detector Conversion Gain

Use a *fixed* operating voltage for your ion detector (multiplier, Daly detector, etc.). The fixed voltage used for measurements within *Part 1* can differ from the fixed voltage used for *Part 2.* But you must use the same fixed voltage for all back-to-back units within any one Part. For each Part, the fixed voltage should provide the best compromise between the *S/N* of A^+^ and the *S/N* of C^+^.

##### 7. Target Gas Purity

The purity of the Ar and SF_6_ target gases should be >99.99%.

##### 8. P/XQQ Measurements

There are only two requirements for P/XQQ measurements: (1) that the sensor being used must provide a *reproducible, single-valued* response for any given target gas pressure of Ar, and (2) that you know the relative sensitivity of the sensor (i.e., its response factor) for SF_6_ relative to Ar. For example, some instruments use a mass flowmeter to measure the flow rate of the target gas into the collision region. You could use the flowmeter response if you know its response factor for SF_fi_ and for Ar.

##### 9. High-Vacuum Materials

No plastic tubing (teflon, etc.) should be used to introduce the high-purity target gas into the collision region within. Q2. The target gas should flow only through materials normally used in high-vacuum applications (bellows valves, etc.). This will ensure that the ln *Y* and ln *W* measurements are not complicated by reactions of the projectile ion with impurities inadvertently admixed with the target gas. If you do have plastic parts in contact with the target gas, and can’t substitute high-vacuum materials for the duration of this test, please mark the top of the first page of the submitted data with the following label: *Plastic Tubing Used.*

##### Test Protocol

*Note:* Before proceeding with the test protocol, please read the *Precautions* above, familiarize yourself with all the steps of the test protocol, and refer to references [9], [12], and [14] as necessary. To ensure consistency among participants, all tuning procedures and measurements must be carried out in accord with the *Precautions.* Please be sure to observe *Precaution 4.* If you have any questions, please call the round-robin coordinator.

*Note:* Record all ion intensities in *relative units (Precaution 6.)* in the spaces provided with each instruction.

##### Part 1. ^36^Ar^+^ + ^40^Ar→^36^Ar + ^40^Ar^+^

*Note:* After you find the tuning conditions that achieve ln *Y*≃ln *W* (*i.e.*, [^40^Ar^+^]≃[^36^Ar^+^]_0_ − [^36^Ar^+^]), the same parameter settings (lens potentials, etc.) must be used for ln *W* measurements as are used for ln *Y* measurements.

*Note:* For instructions 1–7 below, maintain a constant difference of ca. 40 eV between the nominal Ion Source Potential (ISP) and the Q2 Rod Offset (Q2RO); i.e., ISP-Q2RO≃*40 eV.*

*Warning:* If your instrument can only achieve a nominal collision energy which is *less than* 40 eV (LAB), please indicate here your maximum achievable collision energy.

______ eV(LAB)

If you *cannot* achieve 40 eV (LAB), then use your maximum achievable collision energy in lieu of 40 eV for all measurements in Part 1 and Part 2.

##### Initial Tuning

1. PIBON/TGOFF: Set Q1 and Q3 at the mass peak position for ^36^Ar^+^. Measure the ion intensity at the mass peak position (≃[^36^Ar^+^]_0_).
2. Repeat 1., but with PIBOFF/TGOFF to measure the corresponding background intensity.
3. PIBON/TGON: Add sufficient Ar target gas so the ion intensity [^36^Ar+] becomes ca. 0.5 [^36^Ar^+^]_0_.
4. Repeat 3., but with PIBOFF/TGON to measure the corresponding background intensity.
5. PIBON/TGON: Set Q1 at the mass peak position for ^36^Ar^+^ and Q3 at the mass peak position for ^40^Ar^+^. Measure the ion intensity at the mass peak position for ^40^Ar^+^ (≃[^40^Ar^+^]).
6. Vary all the ion-optical elements of your instrument (*q*_2_ lens potentials, rod offsets, resolution and *∆M* control, etc.) as necessary to maximize [^40^Ar^+^] and to roughly approximate [^40^Ar^+^]≃[^36^Ar^+^]_0_ − [^36^Ar^+^].
7. Repeat 1–6 through as many iterations as necessary to achieve [^40^Ar^+^]≃[^36^Ar^+^]_0_ − [^36^Ar^+^].

##### **Q2 Stopping Curve** (cf. *Precaution 3*)

1. PIBON/TGOFF: Set Q1 and Q3 at the mass peak position for ^40^Ar^+^. Measure [^40^Ar^+^]_0_ as a function of the Q2 rod offset (Q2RO) to generate a Q2 stopping curve. Vary Q2RO over a range of potentials from ca. 10 eV below the nominal ion source potential to just above the nominal ion source potential where 99% of the projectile ions are stopped. For each Q2RO used, retune *q*_2_ to $q_{\text{react}}^{\max}$ before measuring each [^40^Ar^+^]_0_ for the corresponding Q2RO. Plot [^40^Ar^+^]_0_ vs Q2RO.
2. If your Q2 stopping curve (energy distribution) doesn’t approximate that of reference [12] (viz. a sharp cutoff), raise the Q1 rod offset closer to the ion source potential. Then repeat 1–7, as necessary, to ensure that the tuning of the ion-optical elements has maintained [^40^Ar^+^]≃[^36^Ar^+^]_0_ − [^36^Ar^+^]. Then repeat 8 (and 9 if necessary).
3. For your final Q2 stopping curve, enter the values of [^40^Ar^+^]_0_ vs the Q2 rod offset (Q2RO) in the spaces provided, and submit a hardcopy of the corresponding plot.

**Table:**

[^40^Ar^+^]_0_	______	______	______	______	______	______	______	______
Q2R0, V	______	______	______	______	______	______	______	______
[^40^Ar^+^]_0_	______	______	______	______	______	______	______	______
Q2R0, V	______	______	______	______	______	______	______	______
[^40^Ar^+^]_0_	______	______	______	______	______	______	______	______
Q2R0, V	______	______	______	______	______	______	______	______

Please submit a record of the settings of all your ion-optical elements (lenses, rod offsets, etc.) used to accomplish 10. Also, please submit a copy of a simplified schematic diagram (layout) showing the ion-optical components of your instrument from the ion source through the detector. Please label each ion-optical element so we can identify the settings with the schematic layout.

*Note:* Use the final Q2 stopping curve developed in instruction 10 to determine *E*_50_ (≡the Q2 rod offset that stops 50% of the projectile ions). For instructions 11–31 below, set the Q2 rod offset= (*E*_50_ − 40) eV and set *all* the other ion-optical elements to the same values as were used for the final Q2 stopping curve of instruction 10.

##### Interference of ^40^Ar^+^ at *m/z* 36 and of ^36^Ar^+^ at *m/z* 40

1. PIBON/TGOFF: Set Q1 at the mass peak position for ^36^Ar^+^ and Q3 at the mass peak position for ^36^Ar^+^ [*Precaution* 6 (i)]. Measure and record the intensity at the mass peak position for ^36^Ar^+^.______
2. Repeat 11, but with PIBOFF/TGOFF to measure the corresponding background intensity.______
3. PIBON/TGOFF: Set Q1 at the mass peak position for ^36^Ar^+^ and Q3 at the mass peak position for ^40^Ar^+^. Measure and record the intensity at the mass peak position for ^40^Ar^+^.______
4. Repeat 13, but with PIBOFF/TGOFF to measure the corresponding background intensity.______
5. PIBON/TGOFF: Set Q1 at the mass peak position for ^40^Ar^+^ and Q3 at the mass peak position for ^40^Ar^+^. Measure and record the intensity at the mass peak position for ^40^Ar^+^.______
6. Repeat 15, but with PIBOFF/TGOFF to measure the corresponding background intensity.______
7. PIBON/TGOFF: Set Q1 at the mass peak position for ^40^Ar^+^ and Q3 at the mass peak position for ^36^Ar^+^. Measure and record the intensity at the mass peak position for ^36^Ar^+^.______
8. Repeat 17, but with PIBOFF/TGOFF to measure the corresponding background intensity.______

##### ln *Y* and ln *W* Measurements

All ln *Y/*ln *W* measurements within this Part 1 should be done at the same time (cf. *Precaution* 6). Record P/XQQ, ln *Y*, ln *W*, ARLEFF/LNY, and ARLEFF/LNW in the spaces provided. Please observe *Precaution* 8..

For [^36^Ar^+^]/[^36^Ar^+^]_0_≃0.5:

1. PIBON/TGOFF: Set Q1 and Q3 at the mass peak position for ^36^Ar^+^. Measure the ion intensity at the mass peak position (≃[^36^Ar^+^]_0_).______
2. Repeat 19, but with PIBOFF/TGOFF to measure the corresponding background intensity.______
3. PIBON/TGON: Add sufficient Ar target gas so the ion intensity becomes [^36^Ar^+^]≃0.5 [^36^Ar^+^]_0_. Measure the ion intensity at the mass peak position (≃[^36^Ar^+^]).______
4. Repeat 21, but with PIBOFF/TGON to measure the corresponding background intensity.______
5. PIBON/TGON: Set Q1 at the mass peak position for ^36^Ar^+^ and Q3 at the mass peak position for ^40^Ar^+^. Measure the ion intensity at the mass peak position for ^40^Ar^+^ (≃[^40^Ar^+^]).______
  **Table:**
  P/XQQ = ______	ln *Y = ______*	ln *W = ______*
  	ARLEFF/LNY = ______	ARLEFF/LNW = ______
6. Repeat 19–23, but with sufficient Ar target gas so that [^36^Ar^+^]/[^36^Ar^+^]_0_≃0.8. Record your measurements in the spaces provided for the corresponding instructions.
  **Table:**
  “19.”______	“20.”______	“21.”______	“22.”______	“23.”______
  P/XQQ=______		In *Y* = ______		In *W* = ______
  		ARLEFF/LNY = ______		ARLEFF/LNW =______
7. Repeat 19–23, but with sufficient Ar target gas so that [^36^Ar^+^]/[^36^Ar^+^]_0_≃0.6. Record your measurements in the spaces provided for the corresponding instructions.
  **Table:**
  “19.”______	“20.”______	“21.”______	“22.”______	“23.”______
  P/XQQ = ______		ln *Y = ______*		ln *W = ______*
  		ARLEFF/LNY = ______		ARLEFF/LNW = **______**
8. Repeat 19–23, but with sufficient Ar target gas so that [^36^Ar^+^]/[^36^Ar^+^]_0_≃0.9. Record your measurements in the spaces provided for the corresponding instructions.
  **Table:**
  “19.”______	“20.”______	“21.”______	“22.”______	“23.”______
  P/XQQ = ______		ln *Y* = ______		ln *W* = ______
  		ARLEFF/LNY = ______		ARLEFF/LNW = ______
9. Repeat 19–23, but with sufficient Ar target gas so that [^36^Ar^+^]/[^36^Ar^+^]_0_≃0.7. Record your measurements in the spaces provided for the corresponding instructions.
  **Table:**
  “19.”______	“20.”______	“21.”______	“22.”______	“23.”______
  P/XQQ = ______		ln *Y* = ______		ln *W* = ______
  		ARLEFF/LNY = ______		ARLEFF/LNW= ______
10. Repeat 19–23 for [^36^Ar^+^]/[^36^Ar^+^]_0_≃0.5:
  **Table:**
  “19.”______	“20.”______	“21.”______	“22.”______	“23.”______
  P/XQQ = ______		ln *Y* = ______		ln W = ______
  		ARLEFF/LNY = ______		ARLEFF/LNW = ______
11. PIBON/TGOFF: Set Q1 and Q3 at the mass peak position for ^36^Ar^+^. Measure the ion intensity at the mass peak position (≃[^36^Ar^+^]_0_).______
12. Repeat 29, but with PIBOFF/TGOFF to measure the corresponding background intensity.______
13. Generate XQQCALPLOT by plotting ARLEFF/LNY vs P/XQQ and ARLEFF/LNW vs P/XQQ on the same graph. Submit a hardcopy of your XQQCALPLOT graph.

*Note:* If the two plots are not superimposable [i.e., same slope and intercept (to within ± 10%)], your instrument HARDWARE does not have a dynamically-correct design (cf. reference [9]). Nonetheless, please proceed with Part 2 to assess other aspects of your instrument design.

##### Part 2. N_2_^+^ + SF_6_→N_2_ + SF_5_^+^

*Note:* In Part 2, you will be tuning you instrument to achieve [SF_5_^+^]≃[N_2_^+^]_0_ − [N_2_^+^]. However, your detector may suffer significant differences in absolute response for SF_5_^+^ vs N_2_^+^ [i.e., the Conversion Gain CG_127_ for SF_5_^+^ (*m/z* 127) may differ substantially from the conversion gain CG_28_ for N_2_^+^ (*m/z* 28)]. You will, therefore, determine CG_127_/CG_28_ at the end of Part 2. In the meantime, observe *Precaution* 6 (iv), and maximize [SF_5_^+^] to approximate as best you can [SF_5_^+^]≃[N_2_^+^]_0_ − [N_2_^+^]. For example, if CG_127_/CG_28_≃0.9, you may find the best you can do is [SF_5_^+^]≃0.8{]N_2_^+^]_0_ − [N_2_^+^]}.

*Note:* Please observe *Precaution* 8. For the sensor you are using to make P/XQQ measurements, record here the type of sensor (Bayard-Alpert Ion Gauge, mass flowmeter, etc.) and its relative sensitivity (i.e., the response factor) for SF_6_ relative to Ar.

Type of senso______ Response Factor (SF_6_/Ar)______

*Note:* For instructions 1–7 below, maintain a constant difference of ca. 40 eV between the nominal Ion Source Potential (ISP) and the Q2 Rod Offset (Q2RO); i.e., ISP-02RO≃40 eV.

*Warning:* If your instrument can only achieve a nominal collision energy which is *less than* 40 eV (LAB), then use your maximum achievable collision energy in lieu of 40 eV, as you did for Part 1.

##### Initial Tuning

1. PIBON/TGOFF: Set Q1 and Q3 at the mass peak position for N_2_^+^ with $q_{2}=q_{\text{react}}^{\max}$. Measure the ion intensity at the mass peak position (≃[N_2_^+^]_0_).
2. Repeat 1, but with PIBOFF/TGOFF to measure the corresponding background intensity.
3. PIBON/TGON: Add sufficient SF_6_ target gas so the ion intensity [N_2_^+^] becomes ca. 0.6 [N_2_^+^]_0_.
4. Repeat 3, but with PIBOFF/TGON to measure the corresponding background intensity.
5. PIBON/TGON: Set Q1 at the mass peak position for N_2_^+^ and Q3 at the mass peak position for SF_5_^+^ (*m/z* 127). Measure the ion intensity at the mass peak position for SF_5_^+^ (≃[SF_5_^+^]).
6. Vary all the ion-optical elements of your instrument ( $q_{2}=q_{\text{prod}}^{\max}$, lens potentials, rod offsets, resolution and ∆*M* control, etc.) as necessary to maximize [SF_5_^+^] and to roughly approximate [SF_5_^+^]≃[N_2_^+^]_0_ − [N_2_^+^].
7. Repeat 1–6 through as many iterations as necessary to optimize [SF_5_^+^]≃[N_2_^+^]_0_ − [N_2_^+^].

##### **Q2 Stopping Curve** (cf. *Precaution* 3)

1. PIBON/TGOFF: Set Q1 and Q3 at the mass peak position for N_2_^+^. Measure [N_2_^+^]_0_ as a function of the Q2 rod offset (Q2RO) to generate a Q2 stopping curve. Vary Q2RO over a range of potentials from ca. 10 eV below the nominal ion source potential to just above the nominal ion source potential where 99% of the projectile ions are stopped. For each Q2RO used, retune *q*_2_ to $q_{\text{react}}^{\max}$ before measuring each [N_2_^+^]_0_ for the corresponding Q2RO. Plot [N_2_+]_0_ vs Q2RO.
2. If your Q2 stopping curve (energy distribution) doesn’t approximate that of reference [14], raise the Q1 rod offset closer to the ion source potential. Then repeat 1–7, as necessary, to ensure that the tuning of the ion-optical elements has maintained [SF_5_^+^]≃[N_2_^+^]_0_ − [N_2_^+^], Then repeat 8 (and 9 if necessary).
3. For your final Q2 stopping curve, enter the values of [N_2_^+^]_0_ vs the Q2 rod offset (Q2RO) in the spaces provided, and submit a hardcopy of the corresponding plot.

**Table:**

[N_2_^+^]_0_	______	______	______	______	______	______	______	______
Q2R0, V	______	______	______	______	______	______	______	______
[N_2_^+^]_0_	______	______	______	______	______	______	______	______
Q2R0, V	______	______	______	______	______	______	______	______
[N_2_^+^]_0_	______	______	______	______	______	______	______	______
Q2R0, V	______	______	______	______	______	______	______	______

Please submit a record of the settings of all your ion-optical elements (lenses, rod offsets, etc.) used to accomplish 10.

*Note:* Use the final Q2 stopping curve developed in instruction 10 to determine *E*_50_ (≡the Q2 rod offset that stops 50% of the projectile ions). For instructions 11–39 below, set the Q2 rod offset = (*E*_50_ − 40) eV and set *all* the other ion-optical elements to the same values as were used for the final Q2 stopping curve of instruction 10.

##### Ion Intensity vs *q*_2_

*Note:* The *q*_2_ values (referenced to *q*_3_) must be varied in small increments to ensure that the measurements made here represent correctly the ion imaging occurring within your Q2Q3 structure (i.e., there may be severe oscillations in the ion intensity as *q*_2_ is varied). These data will be used to make the necessary corrections for differences in the relative reaction pathlength and in relative transmission (cf. reference [14]).

*Note:* For instructions 11–17, verify that Q1 and Q3 are still set at their respective mass peak positions each time a different *q*_2_ value is selected.

1. PIBON/TGOFF: Set Q1 and Q3 at the mass peak position for N_2_^+^. Measure the ion intensity at the mass peak position (≃[N_2_^+^]_0_) as a function of *q*_2_. Use values of *q*_2_ between ca. 0.1 and 0.7. Record your measurements in the spaces provided.
  **Table:**
  [N_2_^+^]_0_	______	______	______	______	______	______	______	______
  *q*_2_	______	______	______	______	______	______	______	______
  [N_2_^+^]_0_	______	______	______	______	______	______	______	______
  *q*_2_	______	______	______	______	______	______	______	______
  [N_2_^+^]_0_	______	______	______	______	______	______	______	______
  *q*_2_	______	______	______	______	______	______	______	______
2. PIBOFF/TGOFF: Measure the corresponding background intensity at just one value of *q*_2_.______
3. PIBON/TGON: Add sufficient SF_6_ target gas so the ion intensity [N_2_^+^] becomes ca. 0.7 [N_2_^+^]_0_. Measure the ion intensity at the mass peak position for N_2_^+^(≃[N_2_^+^]) as a function of *q*_2_. Use values of *q*_2_ between ca. 0.1 and 0.7. Record your measurements in the spaces provided.
  **Table:**
  [N_2_^+^]	______	______	______	______	______	______	______	______
  *q*_2_	______	______	______	______	______	______	______	______
  [N_2_^+^]	______	______	______	______	______	______	______	______
  *q*_2_	______	______	______	______	______	______	______	______
  [N_2_^+^]	______	______	______	______	______	______	______	______
  *q*_2_	______	______	______	______	______	______	______	______
4. PIBOFF/TGON: Measure the corresponding background intensity at just one value of *q*_2_.______
5. PIBON/TGON: Set Q1 at the mass peak position for N_2_^+^ and Q3 at the mass peak position for SF_5_^+^. Measure the ion intensity at the mass peak position for SF_5_^+^ (≃[SF_5_^+^]) as a function of *q*_2_. Use values of *q*_2_ between ca. 0.1 and 0.7. Record your measurements in the spaces provided.
  **Table:**
  [SF_5_^+^]	______	______	______	______	______	______	______	______
  *q*_2_	______	______	______	______	______	______	______	______
  [SF_5_^+^]	______	______	______	______	______	______	______	______
  *q*_2_	______	______	______	______	______	______	______	______
  [SF_5_^+^]	______	______	______	______	______	______	______	______
  *q*_2_	______	______	______	______	______	______	______	______
6. PIBOFF/TGON: Measure the corresponding background intensity at just one value of *q*_2_.______
7. Submit the data and hardcopy plots for [N_2_^+^]_0_ vs *q*_2_, [N_2_^+^] vs *q*_2_, and [SF_5_^+^] vs *q*_2_.

##### ln *Y* and ln *W* Measurements

*Note:* After you have found the tuning conditions that maximize SF_5_^+^ so that [SF_5_^+^]≃[N_2_^+^]_0_ − [N_2_^+^], the same parameter settings (lens potentials, etc.) must be used for ln *W* measurements as are used for ln *Y* measurements. Only *q*_2_ should be varied to tune to $q_{\text{react}}^{\max}$ for ion intensity measurements of N_2_^+^ and to $q_{\text{prod}}^{\max}$ for ion intensity measurements of SF_5_^+^.

All ln *Y*/ln *W* measurements within Part 2 should be done at the same time (cf. *Precaution* 6.). Record P/XQQ, and the other requisite measurements in the spaces provided. Please observe *Precaution* 8.

For [N_2_^+^]/[N_2_^+^]_0_≃0.6:

1. PIBON/TGOFF: Set Q1 and Q3 at the mass peak position for N_2_^+^ with $q_{2}=q_{react}^{\max}$. Measure the ion intensity at the mass peak position (≃[N_2_^+^]_0_).______
2. Repeat 18, but with PIBOFF/TGOFF to measure the corresponding background intensity.______
3. PIBON/TGON: Add sufficient SF_6_ target gas so the ion intensity becomes [N_2_^+^]≃0.6 [N_2_^+^]_0_. Measure the ion intensity at the mass peak position (≃[N_2_^+^]).______ P/XQQ=______.
4. Repeat 20, but with PIBOFF/TGON to measure the corresponding background intensity.______
5. PIBON/TGON: Set Q1 at the mass peak position for N_2_^+^ and Q3 at the mass peak position for SF_5_^+^ with $q_{2}=q_{\text{prod}}^{\max}$. Measure the ion intensity at the mass peak position for SF_5_^+^ (≃[SF_5_^+^]).______
6. Repeat 22, but with PIBOFF/TGON to measure the corresponding background intensity.______
7. Repeat 18–23, but with sufficient SF_6_ target gas so that [N_2_^+^]/[N_2_^+^]_0_≃0.8. Record your measurements in the spaces provided for the corresponding instructions.
  **Table:**
  “18.”______	“19.”______	“20.”______	“21.”______	“22.”______	“23.”______
  P/XQQ = ______					
8. Repeat 18–23, but with sufficient SF_6_ target gas so that [N_2_^+^]/[N_2_^+^]_0_≃0.7. Record your measurements in the spaces provided for the corresponding instructions.
  **Table:**
  “18.”______	“19.”______	“20.”______	“21.”______	“22.”______	“23.”______
  P/XQQ = ______					
9. Repeat 18–23, but with sufficient SF_6_ target gas so that [N_2_^+^]/[N_2_^+^]_0_≃0.9. Record your measurements in the spaces provided for the corresponding instructions.
  **Table:**
  “18.”______	“19.”______	“20.”______	“21.”______	“22.”______	“23.”______
  P/XQQ = ______					
10. Repeat 18–23, but with sufficient SF_6_ target gas so that [N_2_^+^]/[N_2_^+^]_0_≃0.6. Record your measurements in the spaces provided for the corresponding instructions.
  **Table:**
  “18.”______	“19.”______	“20.”______	“21.”______	“22.”______	“23.”______
  P/XQQ = ______					
11. 28. Repeat20.______ and 21.______
12. 29. Repeat 18.______ and 19.______

##### Detector’s Conversion Gain Measurement

You must make conversion gain measurements if your instrument uses analog current measurements. If your instrument uses *true* ion pulse counting (e.g., SCIEX TAGA 6000), conversion gain measurements are *not* needed [i.e., ignore this section (instructions 30–39)].

*Warning:* Some instruments use analog current measurements, but report the ion intensities as ion count rates within their data systems (i.e., the ion currents are converted to equivalent ion count rates via current-to-frequency converters or voltage-to-frequency converters or ?). Such instruments still require conversion gain measurements.

Here you will determine the ratio CG_127_/CG_28_ for the conversion gain of SF_5_^+^ relative to that of N_2_^+^. However, to avoid complicating the conversion gain measurements, do not use SF_5_^+^. Instead, use n-butyl-benzene (*m/z* 134) or other stable compound with a mass close to 127.

1. In general, follow the manufacturer’s recommendations for conversion gain measurements. However, if your detector uses a conversion dynode, please observe the following additional precautions to ensure reproducible results.
  *Note:* When making absolute current measurements with the detector turned on, use all the settings used for instructions 18–29. When you are using the Faraday cup (for absolute ion current measurements), turn off the detector’s high voltage but use all the other settings used for instructions 18–29.
2. Make sure both the N_2_ and the n-butylbenzene are flowing together into the ion source at all times. Use a fixed flow rate. Do 32–39 after the flows have stabilized (constant pressure in the ion source region).
3. PIBON/TGOFF: Set Q1 and Q3 at the mass peak position for N_2_^+^ with $q_{2}=q_{\text{react}}^{\max}$ for *m/z* 28. Leave everything turned on and monitor [N_2_^+^]_0_ until it reaches the final (highest or lowest) stabilized ion current for [N_2_^+^]_0_ (ca. 10–15 minutes).
4. PIBON/TGOFF: Set Q1 and Q3 at the mass peak position for 134^+^ with $q_{2}=q_{\text{react}}^{\max}$ for *m/z* 134. Immediately record the initial absolute current measured for [134^+^] with the detector turned on.
  *I*_detector_=______ for *m/z* 134.
5. Repeat 32.
6. PIBON/TGOFF: Set Q1 and Q3 at the mass peak position for 134^+^ with $q_{2}=q_{\text{react}}^{\max}$ for *m/z* 134. Immediately record the initial absolute current measured for [134^+^] with the Faraday cup.
  *I*_Faraday_=______ for *m/z* 134.
7. The ratio of *I*_detector_/*I*_Faraday_ from 33 and 35 provides an estimate for CG_134_=______.
8. Repeat 32–36, but use *m/z* 28 instead of *m/z* 134 in 33 and 35. For *m/z* 28, *I*_detector_=______ *I*_Faraday_=______ CG_28_=______
9. The values from 36 and 37 provide an estimate for the ratio CG_134_/CG_28_=______.
10. Repeat 32–38 at least one more time to ensure your estimates for CG_127_, CG_28_, and CG_127_/CG_28_ are reproducible.

##### Conclusion

1. For each XQQ instrument, please submit:
  1. this completed test protocol (i.e., with the blank spaces filled in)
  2. a copy of the completed *Questionnaire*
  3. any other data requested herein.

Thank you.

###### Questionnaire

*Note:* Put a large asterisk (*) next to any proprietary (confidential) information which must not be divulged.

Participant’s Name and Address: ______

Phone: ______

What type of XQQ instrument (QQ, QQQ, BEQQ, etc.)? ______

Manufacturer? ______ Model No.? ______

Does your XQQ instrument use a molecular beam target within Q2 (e.g., SCIEX TAGA 6000) or a collision cell enclosure surrounding Q2 (e.g., Finnigan TSQ70)?

Check one: Molecular beam ______ Collision enclosure ______

If a collision cell enclosure surrounds Q2, what is the rectilinear pathlength of the collision region (from the entrance aperture to the exit aperture)? ______cm

##### Quadrupole Rod Assemblies

Is the Q1 quad rod assembly enclosed within a housing (enclosure) or uncovered (nude rods, no housing) within the vacuum chamber? Enclosed ______ Uncovered ______

Is the Q3 quad rod assembly enclosed within a housing (enclosure) or uncovered (nude rods, no housing) within the vacuum chamber? Enclosed ______ Uncovered ______

What is the rectilinear length of the Q2 quad rod assembly? ______cm

What is the field radius *r*_0_ for the Q2 quad rod assembly? ______cm

If cylindrical rods are used for the Q2 quad rod assembly, what is the rod diameter? ______cm

Does each quad rod assembly (Q1, Q2, Q3) have its own separate rf/dc generator (quadrupole controller/power supply)? Yes ______ No ______

If you answered “No”, which ones don’t? Q1 ______ Q2 ______ Q3 ______

What is the frequency of operation for Q2? ______MHz

- if variable frequencies are used for Q2, what nominal frequency was used for Ar^+^? ______MHz for N_2_^+^? ______MHz for SF_5_^+^? ______MHz)

What is the frequency of operation for Q1? ______MHz

- if variable frequencies are used for Q1, what nominal frequency was used for Ar^+^? ______MHz for N_2_^+^? ______MHz for SF_5_^+^? ______MHz)

What is the frequency of operation for Q3? ______MHz

- if variable frequencies are used for Q3, what nominal frequency was used for Ar^+^? ______MHz for N_2_^+^? ______MHz for SF_5_^+^? ______MHz)

##### Interquadrupole Lenses

Answer the questions below with reference to the following simplified schematic:

How many lenses are there betwen the Q1 quad rod assembly and the Q2 quad rod assembly (include the Q1 exit aperture and the Q2 entrance aperture in your count)? ______

What is the nominal diameter (in cm) of the aperture of each of the L12 interquadrupole lenses? L12a ______ L12b ______ L12c ______ L12d ______ L12e ______

If you are using a hybrid XQQ instrument (e.g., BEQQ, etc.), indicate the diameter (in cm) of the Q2 entrance aperture ______.

How many lenses are there between the Q2 quad rod assembly and the Q3 quad rod assembly (include the Q2 exit aperture and the Q3 entrance aperture in your count)? ______

What is the diameter (in cm) of the aperture of each of the L23 interquadrupole lenses? L23a______ L23b ______ L23c ______ L23d ______ L23e ______

##### Modes of Operation

Parent-scan mode: Q3 is set to a fixed mass while Q1 scans over a range of masses; one can thus assess which parent ion masses are the progenitors of a given daughter ion mass.

Daughter-scan mode: Q1 is set to a fixed mass while Q3 scans over a range of masses; one can thus assess which daughter ion masses are the progeny of a given parent ion mass.

For the parent-scan mode, how is the value of *q*_2_ set in your instrument?

Check all that apply:

It can be referenced to *q*_1_? ______
It can be referenced to *q*_3_? ______
Other? (Explain) ______

For the daughter-scan mode, how is the value of *q*_2_ set in your instrument?

Check all that apply:

OIt can be referenced to *q*_1_? ______
OIt can be referenced to *q*_3_? ______
OOther? (Explain) ______

**Comments:**
